# Selection by UV Mutagenesis and Physiological Characterization of Mutant Strains of the Yeast *Saprochaete suaveolens* (Former *Geotrichum fragrans*) with Higher Capacity to Produce Flavor Compounds

**DOI:** 10.3390/jof7121031

**Published:** 2021-11-30

**Authors:** Melissa Tan, Yanis Caro, Alain Shum Cheong Sing, Héloïse Reiss, Jean-Marie Francois, Thomas Petit

**Affiliations:** 1 Laboratoire de Chimie et Biotechnologie des Produits Naturels—CHEMBIOPRO, Université de la Réunion, 15 Avenue René Cassin, CEDEX 9, CS 92003, F-97744 Saint-Denis, France; melissa.tan@univ-reunion.fr (M.T.); yanis.caro@univ-reunion.fr (Y.C.); alain.shum@univ-reunion.fr (A.S.C.S.); heloise.reiss@univ-reunion.fr (H.R.); 2 IUT de La Réunion, Département Hygiène, Sécurité, Environnement (HSE), 40 Avenue de Soweto, CEDEX 9, BP 373, F-97455 Saint-Pierre, France; 3 Toulouse Biotechnology Institute (TBI), UMR-CNRS5504 & UMR-INRA 792, INSA, F-31077 Toulouse, France; 4 Toulouse White Biotechnology, UMS:INRA INSA CNRS, 135 Avenue de Rangeuil, F-31077 Toulouse, France

**Keywords:** yeast, *Saccharomyces cerevisiae*, volatile organic compounds (VOCs), UV, mutant

## Abstract

Yeast volatile organic compounds (VOCs), i.e. low molecular weight organic acids, alcohols and esters, are considered as potential and sustainable sources of natural aromas that can replace commonly used artificial flavors in food and other industrial sectors. Although research generally focuses on the yeast *Saccharomyces cerevisiae*, other so-called unconventional yeasts (NCY) are beginning to attract the attention of researchers, particularly for their ability to produce alternative panels of VOCs. With this respect, a *Saprochaete suaveolens* strain isolated from dragon fruit in Reunion Island was shown to produce α-unsaturated esters from branched-chain amino acids (BCAAs) such as isobutyl, isoamyl or ethyl tiglate, which are rarely found in other yeasts strains. Given that β-oxidation allows the growth of *S. suaveolens* on BCAAs as sole carbon source, we developped a method based on UV mutagenesis to generate mutants that can no longer grow on BCAAs, while redirecting the carbon flow towards esterification of α-unsaturated esters. Among the 15,000 clones generated through UV irradiation, we identified nine clones unable to grow on BCAAs with one of them able to produce eight times more VOCs as compared to the wild-type strain. This higher production of α-unsaturated esters in this mutant strain coincided with an almost complete loss of enoyl-CoA hydratase activity of the β-oxidation pathways and with a twofold increase of acyl-CoA hydrolase with not significant changes in the enzymes of the Ehrlich pathway. Moreover, from our knowledge, it constituted the first example of VOCs enhancement in a microbial strain by UV mutagenesis.

## 1. Introduction

Aroma perception results from a complex mixture of volatile organic compounds (VOCs) that are low molecular weight molecules, which provide the originality in food taste [1]. Until now, some of these aromatic compounds, such as 2-phenylethanol that give the rose-like odor [2], are chemically synthetized because of low-cost production. However, the non-sustainability of chemical processes have raised concerns among consumers that are gradually becoming more aware of natural ingredients, food security and sustainability [3]. The direct extraction of natural aroma from fruits or plants could respond to the consumers’ demand. However, the use of petroleum-based solvents is often required to extract these flavors, which may infringe the natural notion of these products. Recent advances in green extraction techniques methods could solve this issue. For instance, supercritical fluids technology has proven its efficiency on aroma and flavor extraction from hops, vanilla or ginger using the green solvent CO_2_ as fluid. In addition, use of alternative solvent easily evaporable at low temperature (such as propane, butane or dimethyl-ether) using liquefied gas technology can provide aroma with reduced solvent residue [4]. However, the production of these aromas from their natural source depends on the weather conditions of the season, requires large areas of land due to their low concentration of plants, which may reduce the space for other crops with food characteristics. To overcome these limitations, the flavors production catalyzed by microorganisms such as yeast is a promising solution [5], taking into account that microbial cultures are carried out in controlled bioreactors and are independent of the season period. However, this microbial approach presents important bottlenecks. For instance, even if microbial systems can naturally produce flavors of interest, these are generally produced at low yield, low titer and low productivity, which results in high cost of production. These challenges can now be addressed by well-mastered tools and techniques of metabolic engineering. In particular, a metabolic pathway of a specific compound not originally produced by industrially tractable microorganisms such as *S. cerevisiae* or *E. coli* can now be made by applying to the system synthetic biology tools as reviewed in several recent papers [6,7]. A complementary solution is to screen in the still largely unexplored ecosystems for new microorganisms endowed with high and diverse flavoring compounds production.

**Figure 1:**
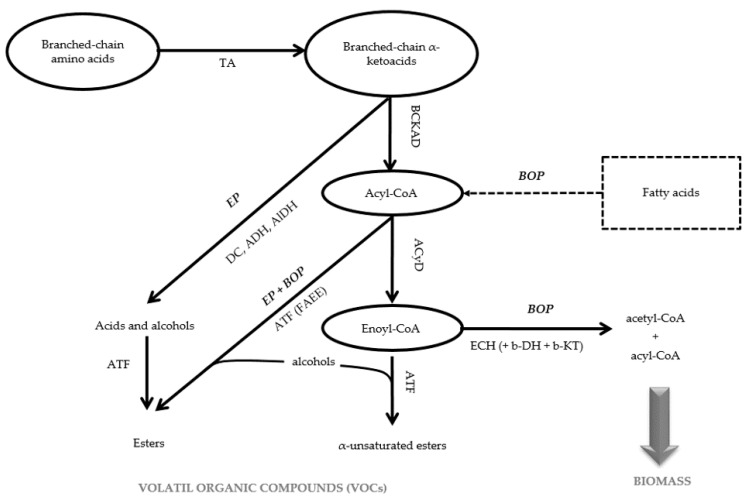
Simplified scheme of catabolism of branched-chain amino acids involving Ehrlich pathway (EP) and β-oxidation pathway (BOP) in *Saprochaete suaveolens.* Abbreviation: TA: transaminase, BCKAD: branched-chain α-keto acid dehydrogenase; ADH: alcohol dehydrogenase; DC: decarboxylase; ACyD: acyl-CoA dehydrogenase-FAD dependent; ECH: enoyl-CoA hydratase; b-DH: β-acyl-CoA dehydrogenase NAD-dependent, b-KT: β-keto acyl-CoA thiolase; AAT: alcohol acyl transferase.

In this regard, non-conventional yeasts (NCY) present interesting flavors production capacities, such as *Pichia kluyveri*, *Torulaspora delbrueckii*, *Candida stellate* and *Hanseniaspora delbrueckii* that were reported to produce high amount of several flavoring compounds like 2-phenyl ethanol, ethyl octanoate, butanoic acid or phenyl esters [5,6,7,8,9,10]. In a previous work, we isolated from dragon fruit in Reunion Island, a filamentous yeast strain with intense fruity flavors production when cultivated on media enriched of amino acids or fatty acids [11]. We identified this yeast species as *Saprochaete suaveolens* by sequencing of the variable D1/D2 domain from nuclear large subunit ribosomal RNA (LSU rRNA) [12]. We then showed that this yeast species produced highly diverse volatile organic compounds (VOCs) among which many α-unsaturated esters (isobutyl tiglate, isoamyl tiglate, butyl tiglate, ethyl tiglate, ethyl 2-methylpropanoate, ethyl-3-methylbut-2-enoate and ethyl but-2-enoate) that are rarely found in aroma bouquet of other *Saccharomyces* and non-*Saccharomyces* species [11,13]. We further demonstrated that the production of these α-unsaturated esters by *S. suaveolens* comes from the catabolism of branched-chain amino acids (BCAAs, isoleucine, leucine and valine) in the β-oxidation pathway (BOP) [14]. A simplified representation of metabolic routes of these BCAAs is presented in Figure 1. 

Briefly, these BCAAs are firstly converted into their corresponding branched α-keto acid in a reaction catalyzed by a branched amino acid transferase (TA). Then, in both *Saccharomyces* and non-*Saccharomyces* species, this α-keto acid enters the Ehrlich pathway (EP) that involves a decarboxylation followed by either an oxidation to produce fusel acids by an aldehyde dehydrogenase (AlDH) or a reduction into fusel alcohols under the action of an alcohol dehydrogenase (ADH) [15]. Esterification of fusel alcohols with acetyl-CoA can generate acetate esters that is catalyzed by an ester synthase or alcohol-O-acetyltransferase (AFT) [16]. A second pathway that has been identified in filamentous fungi but absent in *Saccharomyces cerevisiae* species is the β-oxidation of the branched-chain α-keto-acid into acetyl-CoA [17]. In this pathway, the α-keto-acid intermediate is oxidatively decarboxylated into an acyl-CoA, by the branched-chain α-keto acid dehydrogenase (BCKAD) that is the committed enzyme of this pathway. The produced acyl-CoA can be fully degraded into acetyl-CoA (+ propionyl-CoA in case of L-leucine and L-isoleucine) by the BOP. Ethyl esters can be produced in yeast by condensation of short or medium chain of acyl-CoA derived from fatty acids degradation with ethanol or higher alcohols in a process termed alcoholysis catalyzed by an acyl-CoA: ethanol-acyltransferase (Figure 1) [18,19]. However, a peculiar aspect of *Saprochaete* species is that the enoyl-CoA intermediate deriving from β-oxidation of amino acids can be esterified with alcohols to give rise to various α-unsaturated esters [14]. Altogether, the combination of EP and BOP led to the production of a new variety of VOCs that characterized the aroma bouquet of the *Saprochaete* and *Geotrichum* species [13].

Considering these pathways, one can expect that the inactivation of enzymes catalyzing reactions downstream of enoyl-CoA in the BOP would improve its production of VOCs, while inducing the loss of its ability to grow on media containing only BCAAs as carbon source. Since the 1970s, such inactivation could be attained by targeted genomic alterations using programmable nucleases such as meganuclease, zinc-finger nucleases (ZFNs), transcription activator-like effector nucleases (TALENs) or the CRISPR-Cas9 system (deriving from clustered regularly interspaced short palindromic repeat), which have gained in interest during the last years as they allow single nucleotide changes without introducing DNA double-stranded breaks [20]. However, the use of these directed mutagenesis methods on a specific organism requires the knowledge of the genome sequence. As, the rough genome sequence of *S. suaveolens* has just been released recently [21] and that there were no genetic tools available for this microorganism, we sought to employ a UV mutagenesis method, a random mutagenesis method which enable to enhance the mutation frequency (about 100 fold) of strains without knowing their genome sequence [22]. This technique is particularly interesting as it does not require expensive equipment. Moreover, even if strains produced by UV mutagenesis is recognized as “Genetically modified organism” (GMO) by the European Union legislation (Article 2 of EU Directive 2001/18/EC) [23], they are not concerned by the strict regulation applicable for GMO as they generally have a long-standing safety record [24]. The limitation of UV mutagenesis mostly relies on the need of developing a rapid method of screening to select the potential mutants which display the targeted phenotype among the thousands of cells which have been irradiated. For aroma production, the only screening method which have been described in the literature relies on rapid extraction method (such as solid phase microextraction, SPME) followed by gas chromatography analysis. However, this analysis method is very time consuming and is therefore difficult to directly apply to the thousands irradiated cells generated by UV mutagenis. This is certainly the reason why use of UV mutagenesis to specifically enhance the aroma production of a microbial organism has not been described in the litterature yet. Here, our rational was to first screen for clones that can no longer grow on BCAAs as sole carbon source, since their assimilation required the functioning of BOP (Figure 1), though could possibly assimilate these amino acids through the Erlich or the α-insaturated pathways (Figure 1). Accordingly, nine mutants exhibiting this phenotype were isolated, and their VOCs were quantified by headspace solid phase micro extraction and gas chromatography coupled with mass spectrum detector analysis (HS-SPME GC/MS) after cultivation on agar slants containing glucose and isoleucine. One of them turned out to exhibit the expected phenotype of higher VOCs producer, which is described in this paper.

## 2. Materials and Methods

### 2.1. Yeast Strains, UV Mutagenesis and Screening

The prototroph diploid *Saccharomyces cerevisiae* CEN.PK 112-2N and a *Saprochaete suaveolens* strain (former *Geotrichum fragrans*) previously isolated from Pitaya fruit (*Hylecereus polyrhisus*) in Reunion Island, France [11] were used in this study. UV mutagenesis of *S. suaveolens* was performed according to the protocol described in Winston (2008) [22] established for UV mutagenesis of the yeast *Saccharomyces cerevisiae*, and which was modified for *S. suaveolens*. This strain was incubated in 10 mL of yeast extract peptone dextrose (YPD; 20 g.L^−1^ peptone, 20 g.L^−1^ dextrose and 10 g.L^−1^ yeast extract) medium at 30 °C overnight, collected and washed twice with 10 mL of sterile water. For dose–response assay, an aliquot of 100 µL at 5.10^4^ cell.mL^−1^ was spread on plates containing yeast nitrogen base (YNB) media supplemented of 2 g.L^−1^ of glucose (YNB-Glc) before exposure to UV irradiation (254 nm, 30 cm high) at times ranging from 0 and 540 s. For UV mutagenesis assay, exposure to UV irradiation (254 nm, 30 high) was set at 180 s. To stop photoreactions, plates were kept in the dark and incubated at 30 °C for 15 h until formation of colonies. About 15,000 colonies were obtained from plates after UV mutagenesis assay.

Using replica-plating method, irradiated colonies were replicated on YNB supplemented of 1 g.L^−1^ of either isoleucine, leucine, valine or 20 g.L^−1^ of glucose (YNB-Ile, YNB-Leu, YNB-Val, YNB-Glc respectively) and incubated for 5 days at 30 °C. Colonies growing on YNB-Glc and not on YNB-Ile, YNB-Leu or YNB-Val were then re-streaked on YPD agar media. Confirmation of selected mutant clones was realized by further growth on YPD followed by YNB-Ile. Then, clones were suspended in 5 mL of YPD and incubated overnight at 30 °C. The culture was centrifuged (13,000 rpm, 5 min) and re-suspended in 5 mL of sterile water. Then, 5 µL of 10^6^, 10^5^ and 10^4^ cell.mL^−1^ prepared in sterile water was spotted in duplicate in YNB-agar plate containing glucose, leucine, valine or isoleucine as carbon source. Plates were incubated during 48 h at 30 °C before reading. Wild-types of *S. suaveolens* and *S. cerevisiae* were used as positive and negative control, respectively.

### 2.2. Volatile Organic Compound (VOCs) Semi-Quantitative Analysis

Strains were seeded in a 20 mL crimping vial containing 15 mL of YNB agar slant media supplemented with 2 g.L^−1^ of glucose and 1 g.L^−1^ of isoleucine (YNB-Glc-Ile) and incubated at 30 °C during 24 h. Then, vial was sealed and re-incubated during 24 h at 30°C. Prior to analysis, 10 µL of octan-1-ol (at 1 g.L^−1^ in dichloromethane) was added into the sealed vials as internal standard. The headspace of inclined cultures was subjected to SPME analysis using a 2 cm long fiber coated with 50/30 µm divinylbenzene/carboxen on polydimethylsiloxane bonded to a flexible fused silica core (Supelco). The fiber was exposed to headspace for 15 min at 30 °C and inserted into the injection port at 250 °C for 2 min. Metabolites were separated by Gas Chromatography, on a ZB-5MSI columns (30 m × 0.32 mm × 0.25 µm film thickness), coupled to a mass spectrometer (Shimadzu GCMS-QP2010 Ultra). The carrier gas (H_2_) was set at a flow rate of 2 mL.min^−1^. The column temperature was maintained at 40 °C for 2 min, raised to 150 °C at 10 °C.min^−1^, then raised to 240 °C at 30 °C.min^−1^ before end. Volatile organic compounds were identified by comparing their mass spectra and their experimental Kovats index with the NIST database (www.chemdata.nist.gov). 

### 2.3. Preparation of Crude Extracts and Assays of Enzymatic Activities

Yeast cells were cultivated in 250 mL Erlenmeyer flasks containing 50 mL of YNB-Glc or YNB-Glc-Ile for 24 h at 30 °C. Then, the equivalent of 100 OD units at 600 nm of culture were collected in 50 mL falcon tubes by centrifugation (2000 rpm, 4 °C, 1 min). Cell pellets were re-suspended in 1 mL cold water, transferred into Eppendorf tubes, washed once more with cold water. The pellets obtained after centrifugation (2 min at 10.000 rpm) were stored at −20 °C until use. 

For *S. cerevisiae*, glass beads (0.5 mm of diameter) and 500 µL of extraction buffer (50 mM potassium phosphate buffer, pH 7.4 containing 2 mM EDTA, 100 mM KCl and 1 mM DTT) were added to the cell pellet. Cells were broken in a MP Biomedicals Instrument FastPrep-24^TM^ 5G using six cycles of 30 s at 6.5 m.s^−1^ and 1 min on ice water between each cycle. As this procedure did not work for *S. suaveolens*, these cells were broken using a Qiagen Retsh Tissue Lyser II (3 min, 30 Hz) with a cold tungsten bead introduced in the tube in the presence of 500 µL of the same extraction buffer as above. After centrifugation of the Eppendorf tubes (1000 g, 5 min, 4 °C), the supernatant corresponding to the crude extracts from both yeast species were passed through on Amicon Ultra-05 Centrifugal Filter devices 10 K according to the manufacturer protocol to remove small molecules. Filtered extracts were then used for enzymatic assays and protein determination.

All enzymatic assays were carried out using an Agilent 8453 UV-Visible spectrophotometer in cell cuvettes of 1 mL final volume with an appropriate amount of crude extract. Specificity (or blank) of the enzymatic reaction was checked by carrying out the reaction both in the presence of the substrate without crude extract and in the presence of crude extract without substrates. For glucose-6-phosphate dehydrogenase (G6PDH), the reaction corresponded to the transformation of glucose-6-phosphate into 6-phospho-D-glucolo-1,5-lactone followed by reduction of NADP into NADPH at 340 nm. The reaction mixture that contained 50 mM potassium phosphate buffer (pH 7.4), 1,2 mM EDTA, 120 mM KCl and 2 mM NADP was started by addition of 5 mM glucose-6-phosphate. For the branched-chain α-keto acid dehydrogenase (BCKAD), the reaction corresponded to the oxidative decarboxylation of 2-oxo-4-methylpentanoic acid measured by reduction of NAD^+^ into NADH at 340 nm [25]. The reaction mixture that contained 50 mM potassium phosphate buffer pH 7.4, 2 mM EDTA, 0.2 mM DTT, 0.5 mM TPP, 5 mM MgSO4 and 0.5 mM CoA-SH and 1.5 mM NAD^+^ was started by addition of 0.2 mM 2-oxo-4-methylpentanoic acid. Enoyl-CoA hydratase (ECH) activity was determined according to Park et al., (2003) [26] using 0.225 mM crotonyl-CoA as substrate. The conversion of this substrate into 3-hydroxy-butanoyl-CoA was measured at 263 nm at pH 8.0 in 45 mM Tris-HCl pH 8.0. Decarboxylase (DC) activity was determined using 2-oxo-4-methylpentanoic acid as substrate in a reaction mixture containing 50 mM Na+citrate at pH 6.2, 100 mM KCl, 0.2 mM DTT, 0.5 mM TPP, 5 mM MgSO_4_, 0.2 mM NADH and 5 U/mL yeast alcohol dehydrogenase. The reaction was started by addition of 0.2 mM 2-oxo-4-methylpentanoic acid and oxidation of NAD^+^ was followed at 340 nm. Alcohol dehydrogenase (ADH) was assayed with 2 mM 2-methylpropanal or 100 mM ethanol. In first case, reduction of 0.2 mM NADH to NAD^+^ was followed at 340 nm in a mixture containing 50 mM Na+citrate buffer pH 6.2, 100 mM KCl, 0.2 mM DTT, 0.5 mM TPP and 5 mM MgSO_4_. In the second case, the reaction was carried out in 50 mM potassium phosphate pH 7.4, 100 mM KCl, 0.2 mM DTT, 0.5 mM TPP, 5 mM MgSO_4_ and 0.2 mM NAD^+^. The reaction was started with 100 mM ethanol and oxidation NAD^+^ to NADH was followed at 340 nm. For aldehyde dehydrogenase (AlDH) activity assay, the reaction was carried out in the presence of 50 mM Na+citrate buffer pH 6.2, 100 mM KCl, 0.2 mM DTT, 0.5 mM TPP, 5 mM MgSO_4_ and 1.5 mM NAD+. The reaction was started by addition of 2 mM 2-methylpropanal and followed at 340 nm by reduction of NAD^+^ into NADH. The acyl-CoA dehydrogenase (ACyD) activity was determined following the reduction of DCIP into DCIPH_2_ at 655 nm. The reaction was carried out in 50 mM potassium phosphate buffer pH 7.4, 2 mM EDTA, 100 mM KCl, 0.1 mM FAD^+^, 0.5 mM DCIP and 0.2 mM DTT and was started by the addition of 0.2 mM 2-methylbutanoyl-CoA. The alcohol acyltransferase (AAT) was assayed in the hydrolytic sense (esterase), which has been performed according to Gilham et al., (2005) [27] using p-nitrophenylbutyrate that is hydrolyzed into butyrate and p-nitrophenol, which absorbed at 415 nm. The reaction was carried out in a mixture containing 50 mM potassium phosphate pH 7.4, 2 mM EDTA and 100 mM KCl and started by addition 2 mM p-nitrophenylbutyrate. Finally, acyl-CoA hydrolase activity (ACyH) assay was performed in 10 mM potassium phosphate buffer (pH 6.5), 50 mM MgCl_2_ and 0.1 mM DTNB. The reaction was started by addition of 0.1 mM 2-methylbutanoyl-CoA and the release of CoA-SH was followed at 415 nm, which corresponds to the production of reduced DTNBH. Protein concentration was determined at 550 nm by Bradford method [28] with bovine serum albumin (BSA, Sigma-Aldrich) as the standard. All assays were performed at least in triplicate from independent cultures and for each culture; the essays were performed in duplicate. 

### 2.4. Determination of Strain Growth Rates

Yeast cells were pre-grown in 50 mL tubes containing 5 mL YNB-Glc-Ile overnight at 30 °C. Then, cells were inoculated at OD_600_ 0.1 in 250 mL Erlenmeyer flasks containing 50 mL YNB-Glc-Ile for 8 h at 30 °C. Every hours, OD_600_ of each culture were measured using a spectrophotometer. 

### 2.5. Statistical Analysis

The means and standard deviations (SD) were determined based on triplicate fermentations and represented as mean ± SD. Experimental data were subjected to one-way analysis of variance (ANOVA) using XL Stat Applied Sensory software (3 January 2020) at the 95% confidence level. Using the list of all VOCs identified and quantified by HS-SPME GC/MS in the wild-type and the 9 ILV- mutants of *S. suaveolens*, a principal component analysis (PCA) and a hierarchical cluster analysis (HCA) were applied. PCA and HCA were carried out using the XL Stat Applied Sensory software (3 January 2020) and the data are presented as a biplot graph. A heatmap was elaborated based on the ratio of each VOCs produced by the mutants versus those produced by the wild-type of *S. suaveolens* using NG-CHM Builder: Interactive heatmap online software (www.build.ngchm.net).

## 3. Results and Discussion

### 3.1. Condition of UV Mutagenesis and Screening Strategy

**Figure 2:**
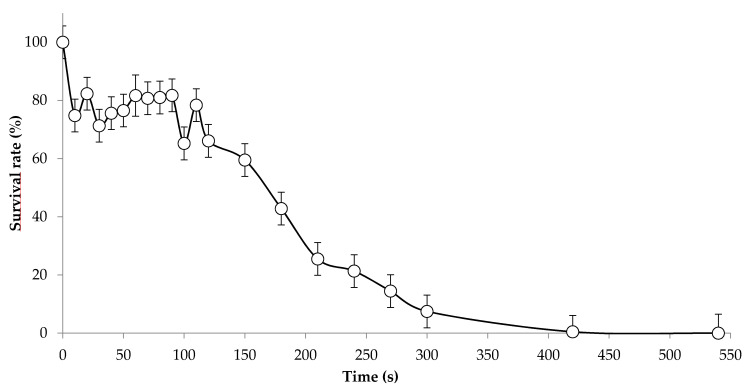
Survival rate of *S. suaveolens* under UV exposure. The values are rom eight independent experiments with standard value presented as vertical bar.

Suitable time of UV irradiation to accumulate non-lethal stable mutations in *S. cerevisiae* has been empirically described as the time that results in a population survival rate ranging from 70 to less than 40% [22]. Therefore, prior to the UV mutagenesis experiment on our *S. suaveolens* wild strain (µ = 0.05 h^−1^), we carried out a dose response assay to determine the optimal UV-exposition duration of this NCY strain. As shown in Figure 2, population survival rate immediately dropped by 25% after the first 10 s of irradiation and then remained at this value even after 110 s of irradiation. Upon longer time of irradiation, we clearly observed a linear decay (R² = 0.96) in population survival with complete mortality after 420 s of irradiation. We, therefore, chose 180 s of UV irradiation, which corresponded to about 50 % of population survival. Accordingly, we isolated 15,393 colonies after this time of UV-irradiation of wild *S. suaveolens* on YNB-Glc agar media plates.

To screen for mutants that have lost of the ability to grow on media containing only BCAAs as source of carbon, around 15,000 irradiated clones were replicated on YNB-Ile, YNB-Leu and YNB-Val agar media plates. Only 10 out of these 15,000 clones showed a loss of ability to grow on these media. These 10 clones were re-cultivated on YNB-Glc agar media to isolate a single colony which was then rechecked on YNB-Ile, YNB-Leu and YNB-Val. According to this procedure, only one was definitively lost. The nine remaining stable *ILV^-^* mutants were further investigated for their growth rate on YNB-Glc and value obtained were comparable to the wild-type (0.05 ± 0.01 h^−1^) except for M6 that showed a µ of 0.001 h^−1^.

### 3.2. Multivariate Analysis of The Wild-Type of S. suaveolens and Its 9 ILV-Mutants Based on Their VOCs Production

**Figure 3:**
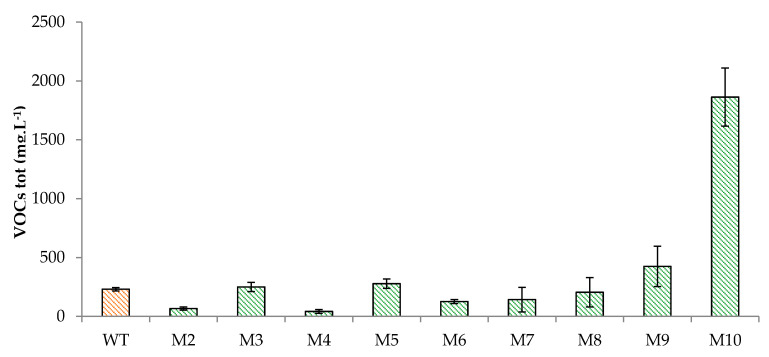
Total VOCs determined by gas chromatography coupled to mass spectrum detector (HS-SPME-GC/MS) analysis in the wild type and the 9 ILV^-^ isolated mutants. Abbreviations: WT, wild-type of *S. suaveolens*; M2, M3, M4, M5, M6, M7, M8, M9, M10, and *ILV*^-^ mutants of *S. suaveolens* produced by UV mutagenesis. Data shown are the mean ± SD of three independent cultures.

Semi-quantitative analysis of the VOCs produced by the wild-type *S. suaveolens* and the 9 ILV^-^ mutants were investigated by headspace-solid-phase microextraction followed by gas-chromatography coupled to mass spectrum detector (HS-SPME-GC/MS) analysis after 48 h of growth on YNB agar tubes containing 20 g.L^−1^ of glucose and 1 g.L^−1^ of isoleucine (YNB-Glc-Ile) at 30 °C. Assuming that the growth of mutants and the wild-type of *S. suaveolens* on agar slants was identical as it was shown on liquid media (see above), we found that mutants M9 and M10 had levels of total VOCs 1.8 and 8 fold higher than the wild-type *S. suaveolens*; whereas VOCs were twofold lower in M6 and M7 and more than 4 fold lower in M2 and M4 mutants (Figure 3). The remaining two mutants M3 and M5 showed roughly a similar level of VOCs as the wild-type. We could notice that the number of different VOCs identified ranged from 32 in the wild-type to 24 in mutant M2 and that there was no correlation between this number and the total amount VOCs produced (Table 1). In addition, our VOCs method being semi-quantitative and limited to a single time point, we may have lost interesting information about the kinetic production of VOCs by each mutant, which will require more sophisticated technology such as a derivative method of Direct-Injection Mass Spectrometric (DIMS) technologies allowing analysis of VOCs detection [29]. However, as a first exploratory and relative fast method, this HS-SPME-GC/MS appears to be reliable. 

**Figure 4:**
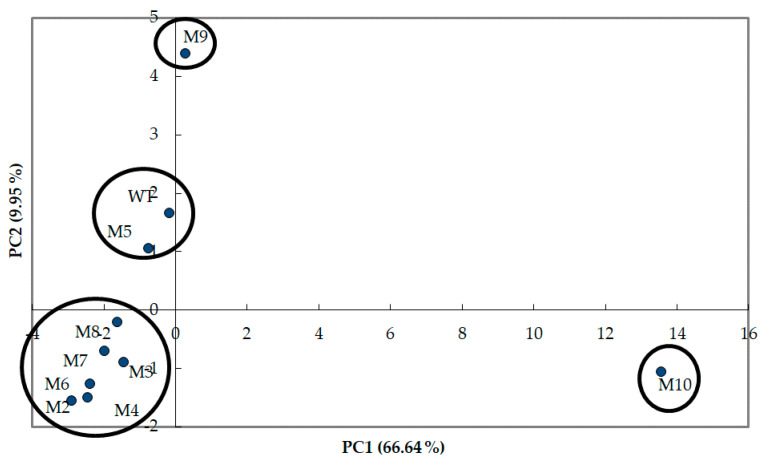
Score plot of PC2 vs PC1 according to the VOCs produced by the wild type and the UV-generated mutants of *S.* suaveolens. PCA was carried out using the Pearson’s method using the mean data of VOCs obtained for each strain. The Cattel’s scree diagram based on the eigenvalues, the cumulative variability, the correlations data between the VOCs and the principle components and the different VOCs contribution to PC_1_ and PC_2_ axis are provided in Figures S1 and S2 and Table S1 respectively.

Using the list of all VOCs identified and quantified in the wild-type and the nine ILV- mutants of *S. suaveolens* in Table 2 a principal component analysis (PCA) and a hierarchical cluster analysis HCA were carried out by the Person’ method. As shown in Figure 4, these statistical tools allowed identifying four groups of strains according to their VOCs profile. From PCA analysis, the first axis PC_1_ that accounted for 66.6% of variance clearly isolated mutant M10 from a group that encompassed the wild-type with mutant M2, M3, M4, M6, M7, and M8. The second PC_2_ axis that accounted for 10% of total variance allowed to separated M9 from WT and mutant M5 which roughly gathered in a same group, indicating that this mutant exhibited a similar VOCs profile as the wild-type. On the other hand, the VOCs profiles of the mutant M9 and M10 were the most different from the wild-type, which is consistent also with the higher amount of VOCs in these mutants. As the PCA analyses show clustering of the M2, M3, M4, M6, M7, and M8 mutants and that this clustering is close to the WT as well as to the M5 mutant, this highlights that the VOC profile of these mutants is very similar to each other and close to that of the wild-type strain. In conclusion, these statistical analysis reinforce the success of our UV mutagenesis in generating at least two interesting mutants exhibiting a VOCs profile quite distinct from the wild-type.

### 3.3. Comparative Analysis VOCs Production between S. suaveolens and the 9 ILV-Mutants

**Figure 5:**
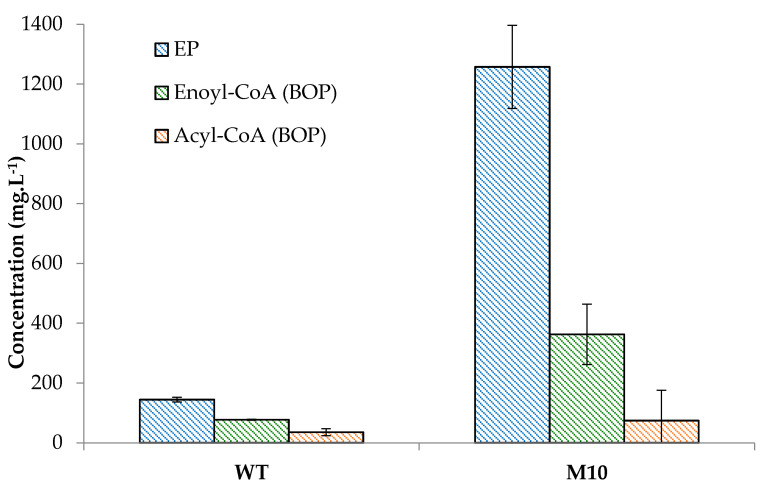
Concentration of esters from Ehrlich pathway (EP), and acyl-CoA and enoyl-CoA from β-oxidation pathway (BOP) in the wild-type and the mutant M10 of *S. suaveolens*. The VOCs were determined after 48 h of growth at 30 °C on YNB-Glu-Ile agar medium. Data shown are the mean ± SD of three independent cultures.

As indicated in Table 2, and already noticed in a previous work [13], the VOCs produced by the wild-type and mutants of *S. suaveolens* were essentially ester compounds. Two types of esters have been reported, namely acetate esters that are formed by condensation of an higher alcohol usually produced from the EP with acetyl-CoA and ethyl esters that are formed by condensing an acyl-CoA with ethanol or another alcohol such as methanol, propanol or butanol [18]. While both types of esters were also found in the VOCs of mutants and the wild-type of *S. suaveolens*, other types of esters compounds were identified. Some of them resulted very likely from the condensation of an alcohol derived from the isoleucine catabolism by the EP (i.e., 2-methylbutanol) with an acyl-CoA (i.e., propanoyl-CoA, butanoyl-CoA), giving rise to 2-methylbutyl butanoate and 2-methylbutyl propanoate. The origin of these short chain acyl-CoAs can be from even carbon chain of fatty acids degradation in the case of butanoyl-CoA or from β-oxidation of isoleucine that leads to propanoyl-CoA and acetyl-CoA. Other esters can be produced from a fusel acid such as 2-methylbutanoate acid that needs to be activated as CoA-intermediates to condense with ethanol and yield ethyl 2-methylbutanoate. These esters were ranged in the EP category due to fusel alcohols and acids that originated from this pathway. Another category is esters that are formed by condensing an acyl-CoA with ethanol or a higher alcohol such as octanol to yield octyl acetate, octyl propanoate and octyl butanoate. We attributed this category to esters from the acyl-CoA of β-oxidation pathway BOP) based acyl-CoA. Finally, a third group that is likely original for *Saprochaete* species included all VOCs whose the acid precursor is an enoyl-CoA that can be esterified with ethanol (i.e., ethyl tiglate, ethyl but-2-enoate) or with a higher alcohol from EP such as 2-methylbutanol and 2-methylpropanol, to yield 3-methylbutyl-2-methylbut-2Z-enoate (isoamyl angelate) and 2-methylpropyl-2-methylbut-2E-enoate (isobutyl tiglate). We have classified these esters in the category of compounds derived from the enoyl-CoA of the BOP (Table 2). Figure 5 illustrated clearly the greater capacity of M10 to produce esters from these three categories, with notably a 5-fold increase of esters from EP, reaching the exceptional level of more than 1 g.L^−1^ and about 10 fold more esters from the enoyl-CoA intermediates. The presence of this new category of esters raises the question of the nature of the ester synthase that can carry out this reaction in *S. suaveolens*. Indeed, so far, an alcohol acetyltransferase encoded by *ATF1/ATF2* [30,31] has been characterized in *S. cerevisiae* as being the major enzyme for the production of acetate esters and an acyl-CoA: ethanol O-acyltransferase encoded by *EHT1/EEB1* was shown to be specific for the synthesis of medium-fatty acid ethyl ester in this yeast species [19,32]. 

**Figure 6:**
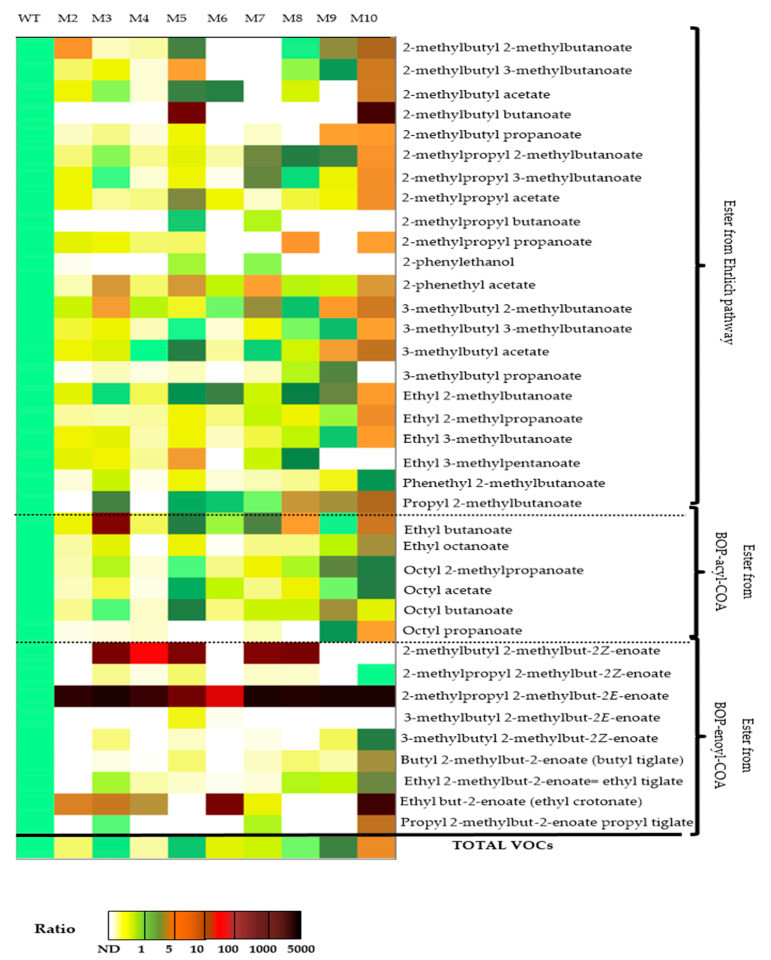
Heatmap representation of VOCs determined in the wild-type and 9 ILV mutants of *S. suaveolens* obtained by UV mutagenesis. Data are represented as ratio of the VOCs value in the mutant versus in the wild-type. VOCs were classified as mainly derived from Ehrlich pathway (EP), from acyl-CoA or enoyl-CoA intermediates of the β-oxidation pathway.

As indicated above, our strategy of selecting strong VOC producers, based on mutants unable to grow on a synthetic medium in which the branched amino acid is the sole source of carbon, has led to the isolation of two mutants M9 and M10 that presented both higher amount and different VOCs profiles than the wild-type of *S. suaveolens*. To further highlight these differences between strains, the content of each VOC in the mutants was divided by that of the wild-type. Then, the resulting ratio value was expressed by a color code ranging from white (absence), green (~1), brown (>10) to dark red (>100), yielding a heatmap shown in Figure 6. This representation clearly reinforced our multivariate analysis indicating that the VOCs profile of the mutant M10 and, to a lesser extent, that of M9 differed from the wild strain. In particular, the VOCs profile in M10 mutant displayed two relevant features. Firstly, levels of 17 out of the 22 VOCs categorized in the EP pathway were 5 to 50 times higher than in the wild strain. More specifically, esters that were produced from alcohol (2-methyl butanol) or acid (2-methylbutanoate) originated from isoleucine catabolism were about 10 times more abundant than in the wild-type. This result could suggest an increased activity of the catabolic activity of isoleucine in the Ehrlich pathway. On the other hand, the production of esters obtained by condensation of acyl-CoA with ethanol and enoyl-CoA with ethanol or EP-derived alcohols (i.e., ethyl tiglate, butyl tiglate, isobutyl tiglate, etc.) were 5 to 100 times higher in this M10 mutant than in the wild-type. This data strongly indicated that an enzymatic reaction downstream of the oxidation of the enoyl-CoA has been altered by UV mutagenesis, leading to higher availability of acyl-CoA and enoyl-CoA intermediates to be esterified with alcohols derived either from isoleucine catabolism or from glycolysis (ethanol). 

The other mutants displayed an overall reduction of VOCs (see Table 1 and Figure 6). In particular, levels of flavors compounds from EP were significantly reduced or even absent as for esters derived from 2-methylbutanol in M6 and M7. In addition, the production of VOCs from the degradation of isoleucine by β-oxidation was lower than in the wild-type of *S. suaveolens* with the exception of ethyl but-2-enoate (ethyl crotonate) which was roughly 3, 10, and 50 fold higher in M4, M2/M3, and M6, respectively (see Table 2) than in the wild-type. This ethyl ester could be produced by condensation of crotonyl-CoA, which may come from the degradation of fatty acids, with ethanol. The same could be true for ethyl butanoate, the amount of which was 10 and 100 times higher in mutants M8 and M3 (see Table 2). Overall, these results suggest that UV mutagenesis has likely altered the ability of these mutants to uptake and assimilate isoleucine, which may be consistent with their inability to grow on a medium that contains only this branched amino acid as a carbon source. 

### 3.4. Loss of Enoyl-CoA Hydratase Activity in the β-Oxidation Pathway May Account for Higher VOCs Production in Mutant M10 

Our finding that the mutant M10 showed a 8-fold increase in VOCs and more specifically that esters derived from condensation of acyl-CoA and enoyl-CoA with alcohols were dramatically increased as compared to the wild-type suggested that activity of enzymes in the β-oxidation that catalyze reactions downstream of enoyl-CoA had been altered. On the other hand, higher levels of EP-derived flavors in M10 may also suggest that the activities of enzymes in the EP had been increased. To address these issues, we decided to determine the activity of key enzymes in both the EP and the BOP. The EP implicates a decarboxylase (DC) and alcohol dehydrogenase (ADH) for the reductive route or an aldehyde dehydrogenase (AlDH) for the oxidative route [15]. The catabolism of branched amino acids by β-oxidation requires a branched-chain α-keto-dehydrogenase (BCKAD) which yields an acyl-CoA. This acyl-CoA intermediate can lose the CoA by an acyl hydrolase (AcyH) to yield the corresponding acid, to be reduced into an enoyl-CoA by a FAD^+^-dependent acyl-CoA dehydrogenase (ACyD) or even esterified into ethyl-esters by a medium-chain fatty acid ester synthase [33]. Specifically, in *S. suaveolens*, enoyl-CoA can be esterified into esters by an alcohol acetyltransferase, the nature of which remains to be identified. 

**Figure 7:**
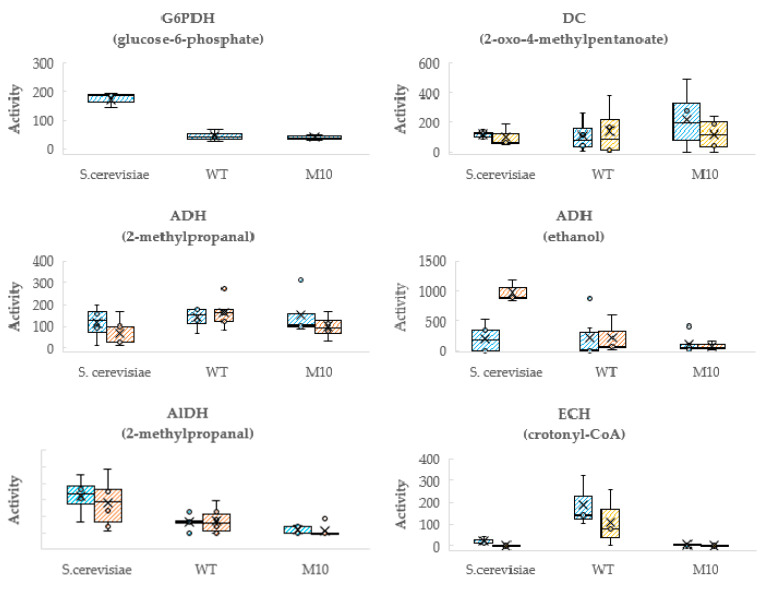

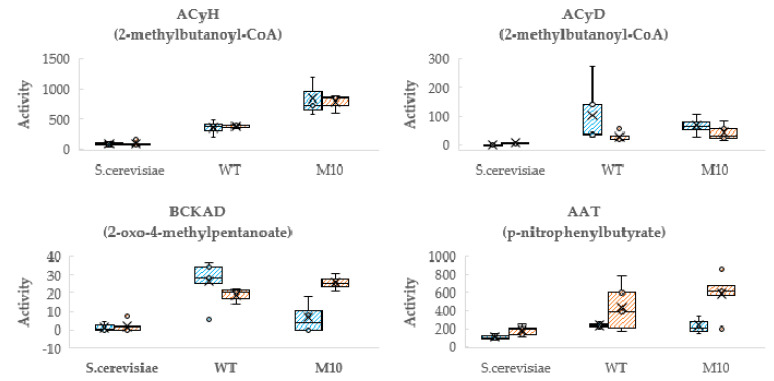
Activities of main enzymes involved in branched amino acids catabolism by the Ehrlich and the β-oxidation pathways in crude extract of the yeast *S. cerevisiae,* in the wild type of *S. suaveolens* (WT) and its mutant M10 cultivated in growth on YNB-Glc (blue) or YNB-Glc-Ile (orange) media. *Enzymatic activities expressed in nmol.min^−1^.mg protein^−1^ were measured in triplicate and value were represented as Box-plot. Values of each replicate in an a assay is represented as a “o”, the mean value as a “Χ”. and median values as the bar through the plot. Abbreviation: ADH: alcohol dehydrogenase; ACyD: acyl-CoA dehydrogenase-FAD dependent; ACyH: acyl-CoA hydrolase; AlDH: aldehyde dehydrogenase; BCKAD: branched-chain α-ketoacid dehydrogenase; DC: decarboxylase; ECH: enoyl-CoA hydratase; AAT: amino acid esterase.

Enzymes in these two metabolic pathways were therefore determined in crude extracts of the wild-type and the mutant M10 of *S. suaveolens*, as well as in the crude extract of *S. cerevisiae* that was used as a control notably for enzymes activity in EP. Results of this experiment are reported in Figure 7. Overall, enzymatic activities measured in crude extracts from the wild-type of *S. suaveolens* and the mutant strains were highly variable from one culture to another. This high dispersion could be due to either the unexpected difficulty of breaking open the cells and obtaining a repeatable cell disruption. However, to ensure that this disruption problem did not prevent the comparison of enzyme activity between the wild type and the mutants, G6PDH activity, which is not expected to be affected by UV irradiation, was measured for both strains growing on YNB-Glc. The results showed good reproducibility of the activity of this enzyme for both the wild type and the M10 mutant, in the range of 45 nmol.min^−1^.mg protein^−1^, which is five times lower than the G6PDH activity measured in *S. cerevisiae* (Figure 7). These data indicate that the process of breaking the cells to prepare the crude extract is reliable. Therefore, we do not yet have a clear explanation for the wide dispersion in the measurement of the activities of the different enzymes of the EP and BOP pathways in both wild type and mutant strains. Despite this problem, statistical analysis of the data allows us to be confident about the enzymatic differences, when they occur, between the mutants and the wild type. Accordingly, and as shown in Figure 7, the DC activity of the wild-type of *S. suaveolens* and mutant M10 was roughly comparable whatever the yeasts were cultivated in YNB-Glc or in YNB-Glc-Ile. Likewise, for the activity of ADH measured on 2-methylpropanal that showed no significant difference between the two strains. We noticed that the AlDH activity was 5 times lower in M10 as compared to the wild-type and overall this enzymatic activity was 10 times lower than that of ADH although levels of VOCs data presented in Table 2 suggested similar contribution of the oxidative and reductive part of the EP in producing the esters. This apparent discrepancy can be due to the fact that the enzymatic measurement in vitro entailed the bulk of ADH activity that may be not relevant to that working in vivo in the EP. 

With respect to enzymes of the BOP, the most dramatic effect was found for the ECH whose the activity was clearly measurable in the WT although showing some dispersion whereas it was almost undetectable in the M10 mutant (Figure 7). We also found that the activity of ACyH that catalyses an upstream reaction of enoyl-hydratase was twofold higher in M10 than in the wild-type. On the other hand, and owing to high variability in enzyme measurement, it can be considered that activity of ACyD and BCKAD in M10 was in the same range of that in the wild-type. On can notice that BCKAD showed the lowest measured activity in yeast crude extract, which seems to agree with previous reports arguing that this enzyme may be rate limiting in the catabolism of branched-chain amino acids by the BOP as it has been reported in mammalian cells [34]. Finally, as we do not know what kind of ester synthase is involved in the formation of esters in *S. suaveolens*, we decided to measure this activity as an “esterase” (EST), which can harbor both an ester synthase and esterase activity [19]. As reported in Figure 7, the global activity of this esterase was similar in both strains but was two-times higher in a glucose synthetic medium supplemented with 1 g.L^−1^ of isoleucine, suggesting a positive effect of the branched amino acids on the expression of the genes encoding this esterase. Taken together, these enzymatic analyses support the idea that the increased VOC production in the *S. suaveolens* M10 mutant can be explained by a loss of ECH activity, which in turn led to an increased availability of the precursors acyl-CoA and enoyl-CoA, which became more available for ester synthesis. In addition, this lack of ECH activity may explain the inability of this mutant to grow on branched-chain amino acids.

## 4. Conclusions

This study aimed at an optimisation of VOCs production and, more specifically, α-insaturated esters by the strain *S. suaveolens*. As genome editing and all the cohorts of molecular techniques currently available for a rational metabolic engineering approaches are not available for this yeast species, including not solely the lack of genome sequence and genetic tools, we have relied on UV mutagenesis to generate mutants that may harbor higher production of VOCs from BCAAs based on the loss of the ability to grow on BCAAs as the sole carbon source, while redirecting the carbon flux from these BCAAs into α-insaturated esters. This strategy turned out to be successful as two out of nine mutants we isolated as unable to grow on this selective medium exhibited the expected phenotype. One of them (the M10 mutant) showed a eight-times higher VOCs production than the wild-type *S. suaveolens*, according to our semi-quantitative detection method. By determining the specific activities of key enzymes involved in isoleucine catabolism, we provided evidence that the higher production of VOCs observed in the M10 mutant was mainly due to loss of the ECH activity and to an increase of ACyH activity. Whether the loss of ECH activity is due to mutation in the gene encoding this enzyme or a regulatory gene shall await the sequencing of the wild-type and the mutant strains.

Nevertheless, this work showed the feasibility of using UV mutagenesis strategy and selection on specific media containing branched-chain amino acids as the sole carbon source to significantly enhance the capacity of the yeast *S. suaveolens* to produce valuable VOCs. Many examples to enhance aroma production in yeasts have been reported in the literature [35,36,37,38,39], but to our knowledge, specific increase of VOCs production by a microbial strain using UV mutagenesis has not been reported, probably because of the lack of an easy and relatively high throughput screening method such as the one we proposed here that relies growth-based phenotype as a first selection step, followed by VOCs measurement of the selected clones. However, this strategy has its limitations as the absence of growth on BCAAs does not necessarily imply an increase in their conversion to flavours. 

## 5. Patents

The work reported in this manuscript was deposited under patent application number: FR2110105. 

## Figures and Tables

**Table 1:** Total number of volatile organic compounds (VOCs) produced by *S. suaveolens* wild type and *ILV-* mutants.

	WT	M2	M3	M4	M5	M6	M7	M8	M9	M10
Total VOCs (mg.L^−1^)	231 ± 14	66. ± 14	251 ± 38	42 ± 16	278 ± 40	126 ± 17	143 ± 104	204 ± 125	424 ± 171	1862 ± 247
Number of total VOCs	34	25	32	29	33	24	31	29	26	31

Abbreviation: WT, wild type of *S. suaveolens*; M2, M3, M4, M5, M6, M7, M8, M9, M10, and ILV- mutants of *S. suaveolens* produced by UV mutagenesis. The VOCs were determined after 48 h of growth at 30 °C on YNB-Glu-Ile agar medium and expressed in mg.L^−1^. Data shown are the mean ± SD of three independent cultures.

**Table 2:** Classification and quantification of VOCs according to pathway production in the wild-type of *S. suaveolens* and its 9 ILV- mutants.

	WT	M2	M3	M4	M5	M6	M7	M8	M9	M10
Ester from the EHRLICH PATHWAY										
2-methylbutyl 2-methylbutanoate	25.8 ± 3.1 ^a^	13.2± 2.9 ^a^	3.4 ± 1.4 ^a^	4.6 ±3.6 ^a^	48.7 ± 25.5 ^a^	0.2 ± 0.4 ^a^	1.6 ± 1.3 ^a^	27.2 ± 46.0 ^a^	74.4 ± 32.5 ^a^	533.2 ± 113.0 ^a^
2-methylbutyl 3-methylbutanoate	5.0 ± 0.3 ^a^	1,5±1,1 ^a^	2.0 ± 3.6 ^a^	0.4 ± 0.3 ^a^	23.7 ± 18.1 ^a,b^	n.d^a^	n.d^a^	4.0 ± 6.9 ^a^	7.0 ± 3.5 ^a^	55.0 ± 30.0 ^b^
2-methylbutyl acetate	4.0 ± 0.8 ^a^	1.8±1.1 ^a^	3.3 ± 2.7 ^a^	0.4 ± 0.2 ^a^	7.8 ± 5.5 ^a^	6.6 ± 2.9 ^a^	n.d^a^	2.3 ± 0.9 ^a^	n.d^a^	42.1 ± 34.0 ^b^
2-methylbutyl butanoate	n.d^a^	n.d^a^	n.d^a^	n.d^a^	0.3 ± 0.1 ^a^	n.d^a^	n.d^a^	n.d^a^	n.d^a^	0.9 ± 0.5 ^b^
2-methylbutyl propanoate	1.0 ± 0.9 ^a^	0.1 ± 0.1 ^a^	0.2 ± 0.4 ^a^	0.1 ± 0.1 ^a^	0.4 ± 0.4 ^a^	n.d^a^	0.1 ± 0.1 ^a^	n.d^a^	4.9 ± 3.0 ^a,b^	6.2 ± 3.5 ^b^
2-methylpropyl 2-methylbutanoate	20.2 ± 4.0 ^a^	5.3 ± 4.9 ^a^	16.7 ± 2.7 ^a^	4.4 ± 1.3 ^a^	10.6 ± 8.4 ^a^	3.6 ± 2.8 ^a^	48.8 ± 69.5 ^a,b^	32.7 ± 21.5 ^a^	37.0 ± 19.0 ^a^	140.5 ± 57.5 ^c^
2-methylpropyl 3-methylbutanoate	2.30 ± 2.6 ^a^	1.0 ± 3.0 ^a^	2.2 ± 1.3 ^a^	0.2 ± 0.3 ^a^	1.0 ± 1.7 ^a^	0.1 ± 0.1 ^a^	5.0 ± 8.4 ^a^	2.6± 1.8 ^a^	1.2 ± 1.0 ^a^	17.1 ± 15.7 ^a^
2-methylpropyl acetate	1.7 ± 0.6 ^a^	0.7 ± 0.9 ^a^	0.3 ± 0.1 ^a^	0.4 ±0.4 ^a^	4.1 ± 2.2 ^a,b^	0.7 ± 0.3 ^a^	0.2 ± 0.1 ^a^	0.6 ± 0.7 ^a^	0.7± 0.3 ^a^	12.0 ± 5.5 ^c^
2-methylpropyl butanoate	0.1 ± 0.1 ^a^	n.d ^a^	n.d ^a^	n.d ^a^	n.d ^a^	0.1 ± 0.0 ^a^	n.d ^a^	0.1 ± 0.0 ^a^	n.d ^a^	n.d ^a^
2-methylpropyl propanoate	1.1 ± 0.6 ^a,b,c^	0.6 ± 0.4 ^a,b,c^	0.4 ± 0.1 ^a,b^	0.3 ± 0.2 ^a,b^	0.3 ± 0.0 ^a^	n.d ^a^	n.d ^a^	7.0 ±2.5 ^d^	n.d ^a^	4.9 ± 2.5 ^c,d^
2-phenylethanol	0.1 ± 0.1 ^a,b^	n.d ^a^	n.d ^a^	n.d ^a^	0.1 ± 0.1 ^a,b^	n.d ^a^	0.1 ± 0.1 ^a,b^	n.d ^a^	n.d ^a^	n.d ^a^
2-phenylethyl acetate	0.2 ± 0.1 ^a^	0.1 ± 0.1 ^a^	0.6 ± 0.8 ^a^	0.1 ± 0.1 ^a^	0.6 ± 0.2 ^a^	0.1 ± 0.2 ^a^	0.7 ± 1.1 ^a^	0.1 ± 0.2 ^a^	0.1 ± 0.1 ^a^	0.6 ± 0.7 ^a^
3-methylbutyl 2-methylbutanoate	6.0 ± 3.0 ^a^	3.8 ± 2.2 ^a^	28.1 ± 25.1 ^a^	4.1 ± 4.0 ^a^	2.3 ± 1.9 ^a^	5.3 ± 5.3 ^a^	18.0 ± 3.6 ^a^	7.4 ± 9.4 ^a^	37.2 ± 40.3 ^a,b^	67.4 ± 34.0 ^a,b^
3-methylbutyl 2-methylpropanotae	2.4 ± 1.2 ^a^	0.8 ± 0.7 ^a^	1.1 ± 1.5 ^a^	0.3 ± 0.2 ^a^	2.4 ± 0.3 ^a^	0.2 ± 0.0 ^a^	1.0 ± 0.9 ^a^	2.0 ± 1.9 ^a^	3.0 ± 1.4 ^a^	12.5 ± 9.3 ^b^
3-methylbutyl acetate	1.2 ± 0.9 ^a^	0.5 ± 0.5 ^a^	0 ± 1.0 ^a^	1.2 ± 1.9 ^a^	2.0 ± 2.2 ^a^	0.3 ± 0.4 ^a^	1.4 ± 0.4 ^a^	0.8 ± 0.8 ^a^	5.3 ± 2.2 ^a,b^	17.4 ± 2.7 ^b^
3-methylbutyl propanoate	0.4 ± 0.4 ^a^	0.1 ± 0.0 ^a^	0.1 ± 0.1 ^a^	0.1 ± 0.0 ^a^	0.1 ± 0.0 ^a^	n.d ^a^	0.1 ± 0.0 ^a^	0.3 ± 0.2 ^a^	0.8 ± 0.9 ^a^	n.d ^a^
ethyl 2-methylbutanoate	28.8 ± 10.6 ^a^	15.60 ± 6.4 ^a^	32.60 ± 5.7 ^a^	9.2 ± 3.5 ^a^	41.0 ± 5.7 ^a^	52.3 ± 10.5 ^a^	17.7 ± 7.0 ^a^	42.8 ± 19.9 ^a^	66.9 ± 31.4 ^a^	171.7± 21.7 ^b^
ethyl 2-methylpropanoate	2.4 ± 0.6 ^a^	0.4 ± 0.3 ^a^	0.4 ± 1.0 ^a^	0.4 ± 0.4 ^a^	0.9 ± 0.7 ^a^	0.6 ± 0.4 ^a^	1.5 ± 0.9 ^a^	1.0 ± 0.9 ^a^	1.7 ± 0.9 ^a^	17.6 ± 3.2 ^b^
ethyl 3-methylbutanoate	9.5 ± 0.7 ^a^	4.3 ± 1.9 ^a^	5.1 ± 0.1 ^a^	1.5 ± 0.9 ^a^	3.9 ± 3.8 ^a^	1.2 ± 0.1 ^a^	3.3 ± 3.0 ^a^	6.3 ± 4.1 ^a^	11.5 ± 6.4 ^a^	56.7 ± 26.0 ^b^
ethyl 3-methylpentanoate	0.1 ± 0.0 ^a^	0.1 ± 0.0 ^a^	0.1 ± 0.1 ^a^	0.1 ± 0.1 ^a^	0.3 ± 0.1 ^a^	n.d ^a^	0.1 ± 0.1 ^a^	0.1 ± 0.2 ^a^	n.d ^a^	n.d ^a^
phenethyl 2-methylbutanoate	1.7 ± 2.0 ^a,b^	0.1 ± 0.1 ^a^	1.0 ± 1.3 ^a^	0.1 ± 0.1 ^a^	0.8 ± 0.5 ^a^	0.1 ± 0.0 ^a^	0.3 ± 0.2 ^a^	0.4 ± 0.5 ^a^	0.7 ± 0.3 ^a^	2.4± 2.4 ^a,b^
propyl 2-methylbutanoate	0.2 ± 0.1 ^a^	n.d ^a^	0.4 ± 0.2 ^a^	n.d ^a^	0.3 ± 0.0 ^a^	0.3 ± 0.0 ^a^	0.2 ± 0.1 ^a^	0.7 ± 0.2 ^a^	0.6 ± 1.0 ^a^	3.8 ± 3.1 ^a,b^
Esters from the Acyl-CoA of BOP										
ethyl octanoate	16.9 ± 12.6 ^a^	3.1 ± 4.2 ^a^	9.0 ± 9.5 ^a^	n.d ^a^	9.2 ± 9.8 ^a^	n.d ^a^	3.9 ± 6.4 ^a^	5.6 ± 9.7 ^a^	11.4 ± 19.0 ^a^	54.5 ± 94.5 ^a^
octyl 2-methylpropanoate	0.7 ± 0.7 ^a,b^	0.1 ± 0.1 ^a,b^	0.5 ± 0.5 ^a,b^	0.1 ± 0.1 ^a^	0.7 ± 0.3 ^a,b^	0.2± 0.0 ^a,b^	0.3 ± 0.1 ^a,b^	0.5 ± 0.0 ^a,b^	1.7 ± 0.6 ^a,b^	1.2 ± 1.0 ^a,b^
octyl acetate	8.60 ± 8.9 ^a^	1.1 ± 0.5 ^a^	2.9 ± 2.4 ^a^	0.5 ± 0.5 ^a^	11.3 ± 6.4 ^a^	5.7 ± 5.4 ^a^	2.0 ± 1.6 ^a^	4.2 ± 4.6 ^a^	7.4 ± 2.4 ^a^	13.9 ± 10.6 ^a^
ethyl butanoate	0.4 ± 0.1 ^a^	0.2 ± 0.1 ^a^	44.9 ± 12.1 ^a^	0.11 ± 0.1 ^a^	0.6 ± 0.4 ^a^	0.3± 0.2 ^a^	0.7 ± 0.7 ^a^	2.2 ± 3.1 ^a^	0.4 ± 0.0 ^a^	4.2 ± 2.8 ^a^
octyl butanoate	9.2 ± 9.5 ^a,b^	2.2 ± 1.9 ^a^	9.4 ± 12.1 ^a,b^	1.2 ± 1.6 ^a^	15.7 ± 10.8 ^a,b^	2.5 ± 0.4 ^a^	6.2 ± 1.9 ^a^	6.2 ± 5.4 ^a^	31 ± 15.4 ^b^	5.4 ± 2.3 ^a^
octyl propanoate	0.1 ± 0.1 ^a^	n.d ^a^	n.d ^a^	0.1 ± 0.1 ^a^	n.d ^a^	n.d ^a^	0.1 ± 0.0 ^a^	n.d ^a^	0.2 ± 0.3 ^a^	0.8 ± 0.7 ^a^
Ester from the Enoyl-CoA of BOP										
2-methylbutyl 2-methylbut-*2Z*-enoate	n.d ^a^	n.d ^a^	0.3 ± 0.20 ^a,b^	0.05 ± 0.06 ^a,b^	0.15 ± 0.01 ^a,b^	n.d ^a^	0.1 ± 0.1 ^a,b^	0.3 ± 0.4 ^a,b^	n.d ^a^	n.d ^a^
2-methylpropyl 2-methylbut-*2E*-enoate	n.d ^a^	3.0 ± 4.9 ^a^	15.9 ± 2.9 ^a,b^	1.9 ± 2.4 ^a^	0.3 ± 0.5 ^a^	0.1 ± 0.1 ^a^	9.4 ± 4.8 ^a,b^	4.1 ± 5.1 ^a^	8.8 ± 5.4 ^a,b^	37.4 ± 20.0 ^c^
2-methylpropyl 2-methylbut-*2Z*-enoate	1.1 ± 0.0 ^a^	n.d^a^	0.2 ± 0.2 ^a^	0.1 ± 0.0 ^a^	0.3 ± 0.1 ^a,b^	n.d ^a^	0.1 ± 0.1 ^a^	0.1 ± 0.0 ^a^	n.d ^a^	1.1 ± 0.7 ^a,b^
3-methylbutyl 2-methylbut-*2E*-enoate	0.2 ± 0.2 ^a^	n.d ^b^	n.d ^b^	n.d ^b^	0.1 ± 0.1 ^a,b^	n.d ^b^	n.d ^b^	n.d ^b^	n.d ^b^	n.d ^b^
3-methylbutyl 2-methylbut-*2Z*-enoate	0.4 ± 0.5 ^a^	n.d ^a^	0.1 ± 0.1 ^a^	n.d ^a^	0.1 ± 0.1 ^a^	n.d ^a^	0.1 ± 0.0 ^a^	n.d ^a^	0. 2 ± 0.2 ^a^	0.6 ± 1.0 ^a^
butyl 2-methylbut-2E-enoate	28.5 ± 2.2 ^a^	n.d ^b^	1.7 ± 1.4 ^b^	0.5 ± 0.7 ^b^	7.7 ± 8.5 ^a,b^	0.1 ± 0.1 ^b^	3.3 ± 4.8 ^b^	7.9 ± 9 ^a,b^	4.9 ± 3.1 ^a^	90.2 ± 10.5 ^c^
ethyl 2-methylbut-2 E-enoate	48.4 ± 3.0 ^a,b^	n.d ^a^	36.0 ± 7.0 ^a,b^	8.3 ± 10.2 ^a^	5.5 ± 2.0 ^a^	1.5 ± 1.4 ^a^	3.5 ± 1.7 ^a^	34.1± 20.4 ^a,b^	32.0 ± 38.5 ^a,b^	115.9± 30.7 ^a,b^
ethyl but-2E-enoate	0.1 ± 0.1 ^a^	1.4 ± 1.4 ^a^	1.7 ± 1.9 ^a^	0.5 ± 0.7 ^a^	n.d ^a^	36.6 ± 0.7 ^a,b^	0.1 ± 0.1 ^a^	n.d ^a^	n.d ^a^	155.8 ± 136.0 ^b^
propyl 2-methylbut-*2E*-enoate	0.1 ± 0.0 ^a^	n.d ^a^	0.1 ± 0.1 ^a^	n.d ^a^	n.d ^a^	n.d ^a^	0.1 ± 0.1 ^a^	n.d ^a^	n.d ^a^	1.0 ± 1.2 ^b^

Abbreviation: WT, wild type of *S. suaveolens*; M2, M3, M4, M5, M6, M7, M8, M9, M10, and ILV- mutants of *S. suaveolens* produced by UV mutagenesis; n.d, non detected.The VOCs were determined after 48 h of growth at 30 °C on YNB-Glu-Ile agar medium, expressed in mg.L^−1^ and classified according to their hypothetical metabolic pathway of production schematically illustrated in Figure 1. Data shown are the mean ± SD of three independent cultures. Groups a, b and c were statistically determined by one-way analysis of variance (ANOVA) using XL Stat Applied Sensory software (3 January 2020) at the 95% confidence level for each volatile, using the amount of 0 mg.L^−1^ when the volatile was not detected for the strains.

## Data Availability

Not applicable.

## Supplementary Materials

The following are available online at www.mdpi.com/article/10.3390/jof7121031/s1, Figure S1: Cattell’s scree diagram (A) based on the eigenvalues and the cumulative variability (B) obtained for principal component analysis of VOCs produced by the wild-type and the UV-generated mutants of S. suaveolens, Figure S2. Different VOCs contribution to PC1 and PC2 axis obtained for principal component analysis of VOCs produced by the wild-type and the UV-generated mutants of S. suaveolens; Table S1: Correlation data between VOCs and principle component obtained for principal component analysis of VOCs produced by the wild-type and the UV-generated mutants of S. suaveolens.
