# Supplementary material for: Selection by UV Mutagenesis and Physiological Characterization of Mutant Strains of the Yeast Saprochaete suaveolens (Former Geotrichum fragrans) with Higher Capacity to Produce Flavor Compounds
Source: J Fungi (Basel). 2021 Nov 30;7(12):1031. doi: 10.3390/jof7121031 (PMC8704521; doi:10.3390/jof7121031)
Supplement: Supplementary file 1 [file jof-07-01031-s001.zip › jof-1446975-supplementary.pdf]

Supplementary data

Figure S1. Cattell's scree diagram (A) based on the eigenvalues and the cumulative variability (B) obtained for principal component analysis of VOCs produced by the wild-type and the UV-generated mutants of *S. suaveolens*

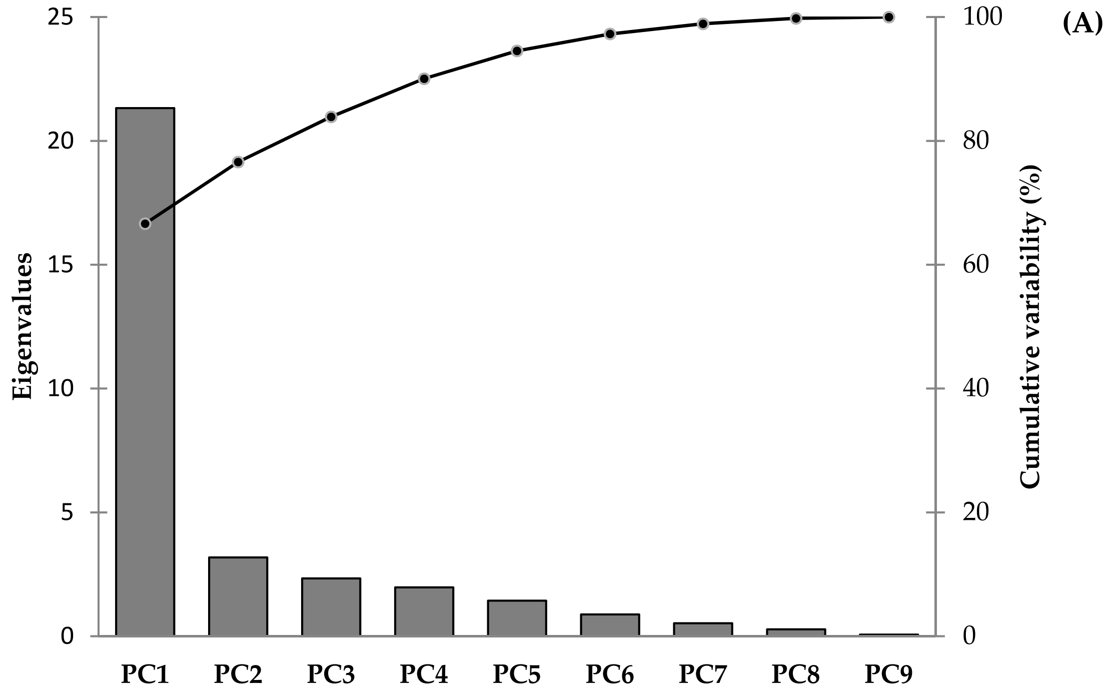

(B)

	PC1	PC2	PC3	PC4	PC5	PC6	PC7	PC8	PC9
**Eigenvalues**	21,325	3,184	2,335	1,970	1,435	0.879	0.523	0.280	0.068
**Variability (%)**	66,641	9,951	7,297	6,157	4,484	2,747	1,635	0.875	0.212
**Cumulative variability (%)**	66,641	76,593	83,890	90.047	94,531	97,278	98,913	99,788	100.000

Table S1. Correlation data between VOCs and principle component obtained for principal component analysis of VOCs produced by the wild-type and the UV-generated mutants of *S. suaveolens*

**ID n°**	**Compound**	**PC1**	**PC2**	**PC3**	**PC4**	**PC5**
**1**	2-phenylethanol	-0.134	0.257	0.788	-0.013	0.073
**2**	2-methylbutyl 2-methylbut-2Z-enoate	-0.278	-0.149	0.102	0.820	0.159
**3**	2-methylbutyl 2-methylbutanoate	0.925	-0.152	-0.100	-0.083	-0.183
**4**	2-methylbutyl 3-methylbutanoate	0.939	0.003	0.200	0.122	-0.223
**5**	2-methylbutyl acetate	0.962	-0.210	0.088	-0.024	-0.034
**6**	2-methylbutyl butanoate	0.941	-0.127	0.173	0.094	-0.221
**7**	2-methylbutyl propanoate	0.841	0.425	-0.304	-0.085	-0.082
**8**	2-methylpropyl 2-methylbut-2E-enoate	0.886	-0.152	-0.285	0.266	0.088
**9**	2-methylpropyl 2-methylbut-2Z-enoate	0.718	0.076	0.478	-0.098	0.416
**10**	2-methylpropyl 2-methylbutanoate	0.940	-0.021	-0.090	0.040	0.000
**11**	2-methylpropyl 3-methylbutanoate	0.943	-0.187	0.001	0.052	0.034
**12**	2-methylpropyl acetate	0.954	-0.090	0.203	0.032	-0.165
**13**	2-methylpropyl butanoate	-0.199	0.002	0.315	-0.551	0.522
**14**	2-methylpropyl propanoate	0.979	-0.019	-0.071	0.089	-0.143
**15**	3-methylbutyl acetate	0.980	0.052	-0.127	-0.022	-0.120
**16**	3-methylbutyl propanoate	-0.054	0.921	-0.260	-0.119	0.160
**17**	butyl 2-methybut-2E-enoate	0.972	-0.052	0.145	-0.094	0.114
**18**	ethyl 2-methybut-2E-enoate	0.918	0.082	-0.040	0.064	0.336
**19**	ethyl 2-methylbutanoate	0.955	0.065	-0.101	-0.030	-0.032
**20**	ethyl 2-methylpropanoate	0.990	-0.110	-0.005	-0.065	-0.009
**21**	ethyl 3-methylbutanoate	0.995	-0.026	-0.064	-0.031	0.020
**22**	ethyl 3-methylpentanoate	-0.161	0.186	0.746	0.435	-0.387
**23**	ethyl but-2E-enoate	0.935	-0.257	-0.052	-0.144	-0.022
**24**	ethyl butanoate	-0.025	-0.182	-0.254	0.723	0.514
**25**	ethyl octanoate	0.987	0.052	0.071	0.031	0.103
**26**	octyl 2-methylpropanoate	0.583	0.785	-0.146	0.135	0.014
**27**	octyl acetate	0.746	0.392	0.432	0.002	-0.056
**28**	octyl butanoate	0.042	0.944	-0.153	0.224	-0.122
**29**	octyl propanoate	0.983	0.073	-0.093	-0.120	0.021
**30**	phenethyl 2-methylbutanoate	0.840	0.180	0.250	0.104	0.388
**31**	propyl 2-methylbut-2E-enoate	0.978	-0.197	-0.022	-0.010	0.024
**32**	propyl 2-methylbutanoate	0.980	-0.084	-0.089	0.032	-0.002

Figure S2. Different VOCs contribution to PC1 and PC2 axis obtained for principal component analysis of VOCs produced by the wild-type and the UV-generated mutants of *S. suaveolens*

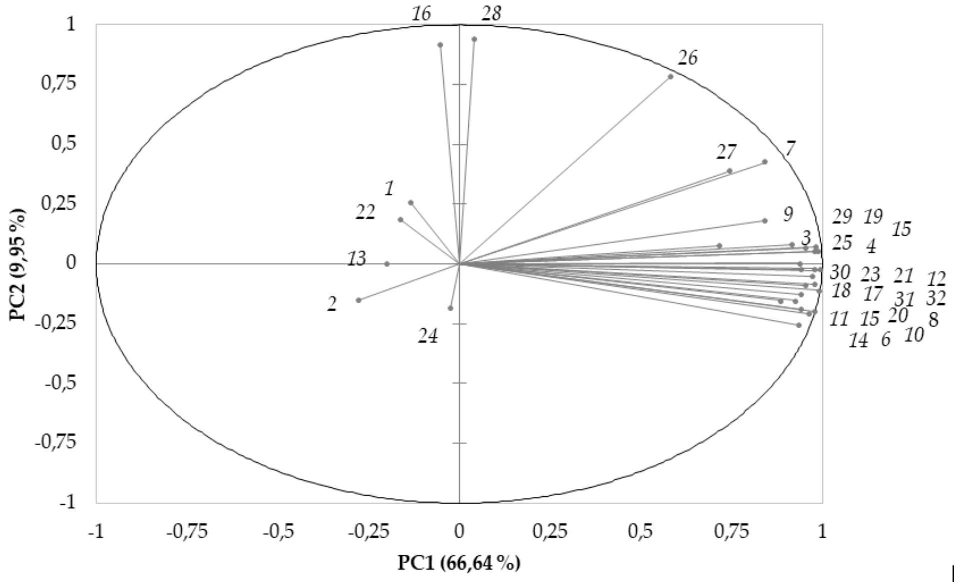

<u>VOCs ID number :</u> **1:** 2-phenyethanol ; **2:** 2-methylbutyl 2-methylbut-2*Z*-enoate; **3:** 2-methylbutyl 2-methylbutanoate ; **4:** 2-methylbutyl 3-methylbutanoate ; **5**: 2-methylbutyl acetate ; **6**: 2-methylbutyl butanoate ; **7**: 2-methylbutyl propanoate ; **8:** 2-methylpropyl 2-methylbut-2E-enoate ; **9 :** 2-methylpropyl 2-methylbut-2Z-enoate ; **10 :** 2-methylpropyl 2-methylbutanoate ; **11 :** 2-methylpropyl 3-methylbutanoate ; **12 :** 2-methylpropyl acetate ; **13 :** 2-methylpropyl butanoate ; **14 :** 2-methylpropyl propanoate ; **15 :** 3-methylbutyl acetate ; **16 :** 3-methylbutyl propanoate ; **17 :** butyl 2-methybut-2E-enoate ; **18 :** ethyl 2-methybut-2E-enoate ; **19 :** ethyl 2-methylbutanoate ; **20 :** ethyl 2-methylpropanoate ; **21 :** ethyl 3-methylbutanoate ; **22 :** ethyl 3-methylpentanoate ; **23 :** ethyl but-2E-enoate ; **24 :** ethyl butanoate ; **25 :** ethyl octanoate ; **26 :** octyl 2-methylpropanoate ; **27 :** octyl acetate ; **28 :** octyl butanoate ; **29 :** octyl propanoate ; **30 :** phenethyl 2-methylbutanoate ; **31 :** propyl 2-methylbut-2E-enoate ; **32 :** propyl 2-methylbutanoate
